# Birthing Unit Closures: Impacts and Opportunities in Iowa

**DOI:** 10.23889/ijpds.v9i5.2879

**Published:** 2024-09-18

**Authors:** W. Todd Abraham, Heather Rouse, Cora Herbkersman, Gio Chighladze

**Affiliations:** 1 Iowa State University

## Objective and Approach

Iowa, US has experienced the closure of 41 birthing units in local hospitals since 2000, which is nearly 50% of previously available units. Public health concern about access to prenatal care and birthing support in this predominately rural state has raised attention to the value of using integrated administrative data to understand impacts and direct needed resources. This presentation will showcase a series of analyses using vital statistics birth records, hospital closure records, and statewide home visiting records to understand (a) closure impacts on relevant subgroups and (b) opportunities to strengthen outcomes through service improvements. Results suggest indirect relationships between hospital closure and child/mother outcomes through impacts on prenatal care. Some mothers (e.g., mothers with low maternal education, older mothers, and mothers with previous children) were more susceptible to negative impacts on prenatal care. Data about mother connections with other public service systems (e.g., Medicaid or WIC) suggested buffering effects for some women who were at risk for birthing unit closure impacts. Subsequent phases of research have used integrated administrative data to highlight hospital closure areas where women differentially experienced more positive outcomes to inform community outreach and identification of effective intervention efforts.

## Methods

This Conclusions and Implications highlights the value of longitudinal administrative data utilized to address policy-relevant topics in public health. Results suggest opportunities to expand and replicate rural health practices that are working to mitigate risk in geographic areas where women face challenging access to a birthing hospital.

